# Global survey to assess preferences when attending virtual orthodontic learning sessions: optimising uptake from virtual lectures

**DOI:** 10.1186/s40510-021-00390-4

**Published:** 2021-12-21

**Authors:** Samer Mheissen, Mohammed Almuzian, Mark B. Wertheimer, Haris Khan

**Affiliations:** 1 Orthodontic Department, Syrian Ministry of Health, Private Practice, Damascus, Syria; 2 University of Edinburgh, Edinburgh, UK; 3 Private Practice, Johannesburg, South Africa; 4 CMH Institute of Dentistry, Lahore, National University of Medical Sciences, Lahore, Punjab Pakistan; 5 Present Address: Irbid, Jordan

**Keywords:** Online, Webinar, Teaching, Zoom, Social, Pandemic

## Abstract

**Objectives:**

Understanding the issues concerning the conducting of virtual orthodontic learning sessions (VOLSs) is essential. This study aims to identify attendees- and host-related aspects that could optimise learning and uptake from the VOLSs.

**Methodology:**

Fourteen pre-validated questions were anonymously and electronically sent to 3000 orthodontic residents and specialists globally. The survey included demographic questions and questions to gauge attendees' engagement, memorising, and motivation-related factors. Reminders were sent at two-week intervals to non-respondents. The survey was closed when the sample size was met. Descriptive and inferential statistics were performed.

**Results:**

593 orthodontic residents and specialists (294 males and 299 females), primarily junior orthodontists and residents aged between 25 and 35 years of age, completed the survey. Post-VOLS recording was highly requested by the participants (8.84/10, 95% CI 8.67–9.00) with no significant influence of demographics on this trend (*p* > 0.05). Most of the participants were in favour of short post-VOLSs feedback (6.79/10 95% CI 6.58–6.99) with significant differences (*p* = 0.048) between participants from different regions of the world. The average number of screenshots taken was 6.1 per lecture. The learners’ interests in attending on-line lectures were mainly to learn new clinical orthodontic tips (96.8%).

**Conclusion:**

Implementing a short feedback survey after VOLSs, the provision of recording, and the provision of certificates of attendance need to be considered.

## Introduction

Healthcare professionals need to periodically update their knowledge and skills by participating in continuous medical education, or continuous professional development (CPD) activities which are traditionally held through face-face lectures, seminars, or workshops. With increased technological advancements, Internet use is becoming the primary source of seeking clinical and theoretical knowledge [1, 2]. This has led to the increased popularity of online CPD activities [3]. Moreover, the COVID-19 global pandemic and its resultant lockdown have increased dependency on online activities [4]. In orthodontics, electronic (e) learning is an established practice at the institutional level for both under- and postgraduate education [5], but was utilised sparingly before the COVID-19 pandemic era to conduct international conferences and CPD activities [4, 6].

Webinars are virtual learning sessions based on information and communications technologies and are established e-learning methods in medical education [7]. These web-based lectures are delivered and attended using computers and mobile devices, and at present several platforms are available to conduct these online sessions. These virtual activities offer the advantage of distance learning, cost-effectiveness, and flexibility with respect to time and place [8]. On the downside, information technology (IT) infrastructure and its related skills are required for conducting webinars. Besides, there is a lack of appropriate culture for this mode of education [7]. Therefore, hosts of online learning sessions need to be familiar with how content should be shared, and how lectures could be made more interactive.

An excellent online session should have the same goals as conventional learning activities in optimising the learning experience and enhancing retention of the concepts [9, 10]. Participants' interaction is a key criterion for accreditation of a webinar [11]. The intra-lecture chat functionality and questions/answers session of the webinar may provide an invaluable opportunity for participant interaction [7]. Interactive polls are another tool for interactions with the audience. A recent study suggested that webinars solving daily clinical questions increase participants' uptake [12]. Other crucial elements to improve the learning experience are the pre-webinar orientation of the hosting platform and implementing clinical case discussions combined with informal quizzes [8].

There is still considerable room for improvement of the quality of webinars and the knowledge uptake from these virtual sessions. Also, there is age and regional diversity in seeking online education [13]. Therefore, this global cross-sectional study was intended to assess the preferences of orthodontic clinicians while attending virtual lectures, and how the uptake of knowledge can be improved from these sessions.

## Methodology

### Study sample size

SurveyMonkey calculator (www.surveymonkey.co.uk) was utilised to calculate the sample size of this study. Considering that the approximate number of on-line orthodontic learners is 25,000, and to detect a 95% confidence level with a margin of error of 4%, the required sample size was 560 participants.

### On-line survey

Eight experts participated in content validity [14, 15]. According to Lawshe’s method [16], the CV ratio (CVR) was calculated, and *n* = 14 questions carried a CVR of more than 0.51. These questions were then sent to 100 residents and specialist orthodontists for face validity [17]. All the questions achieved more than 75% of inter-agreement and were included in the final survey. Both surveys were anonymous. Electronic reminder notices were sent after a week to non-responders.

The final anonymous survey link was sent to 3000 orthodontic residents and specialists. At the start of the survey, details about the study objectives and the researcher team were provided. Reminders were sent at two-week intervals to non-respondents. The survey remained open until the pre-determined sample size was reached. The set of questions was a mix of 10-point scale and multiple-choice questions (Appendix 1).

### Statistical analysis

Descriptive statistics regarding age, gender, experience, and continent were reported. The Kolmogorov–Smirnov test revealed that the data were not normally distributed.As such, Mann Whitney and Kruskal Wallis tests were used for continuous data, while  X2  and Fisher exact tests were used for categorical data. The data were presented as a mean, with a 95% confidence interval (CI). A probability (*p*) value equal to or less than 0.05 was considered statistically significant. All statistical analyses were performed using SPSS software version 25.0 and R Software version 4.0.3 (R Foundation for Statistical Computing, Vienna, Austria).

## Results

### Questions related to participant demographics

**Fig. 1.**
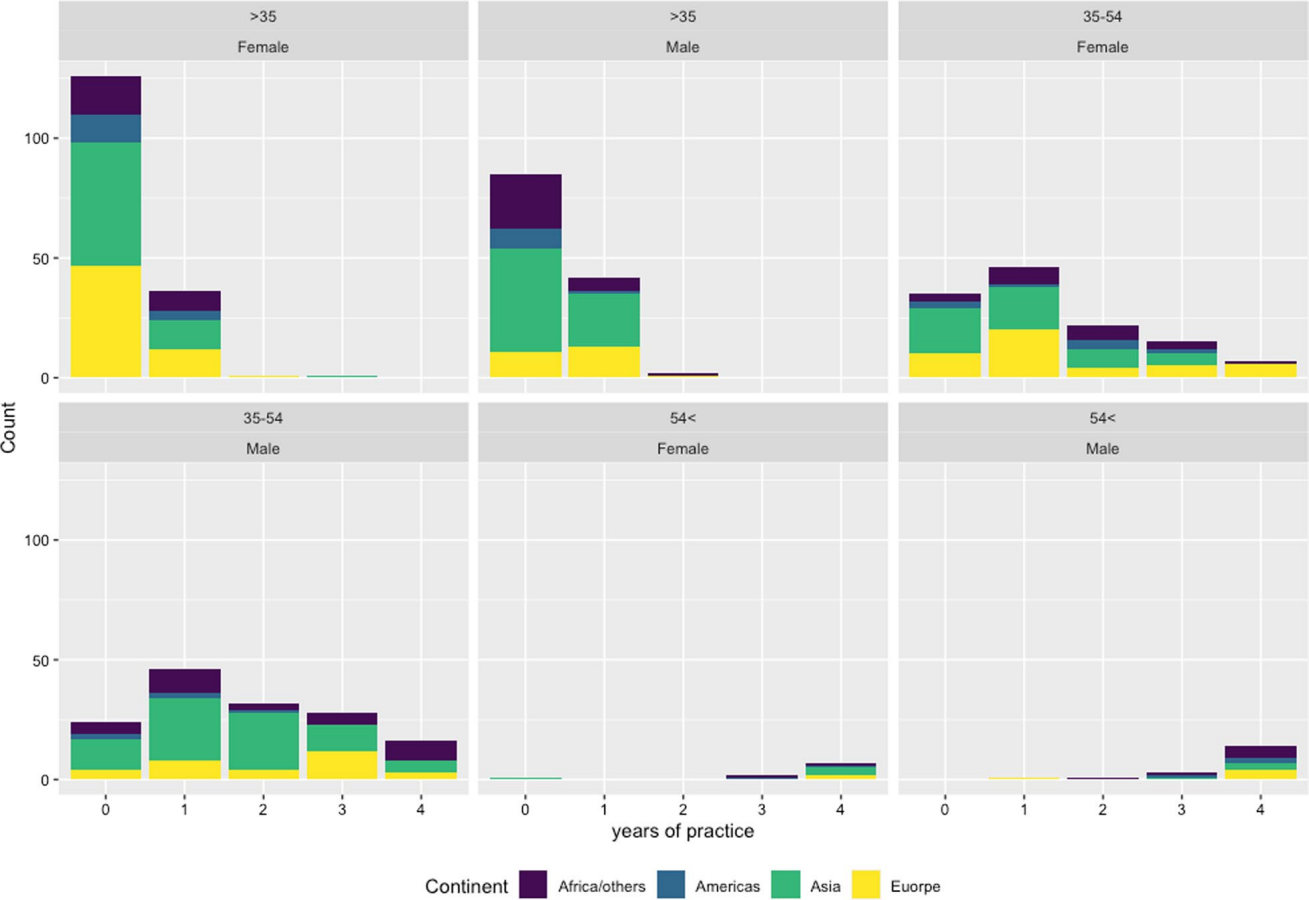
A bar plot showing the survey participants' characteristics according to age, gender, and geographic location

593 orthodontic specialists and residents completed the survey (response rate of 19.9%). 294 (49.6%) were males, and 299 (50.4%) were females. Almost half of the participants (*n* = 293, 49.4%) were between 25 and 35 years of age, 45.7% aged 35–54 years, while the rest (4.9%) was from the old age group (older than 54 years). Nearly half of the participants (54.1%) were junior orthodontists or residents in orthodontics with less than 5 years of experience. Most of the participants were from Asia (51.4%) followed by Europe (22.4%), Africa (18.2%), North America (5.6%), South America (1.3%), and Australasia (1.0%) (Fig. 1 and Table 1).

**Table 1:** Descriptive data about the participants

Gender (number and percentage)	Age (number and percentage)	Years of experience (number and percentage)	Continent (number and percentage)
294 males, 49.6%	Less than 35 years (young participants) 272 participants, 45.9%	Less than 5 years 272, 45.9%	Asia 305, 51.4%
299 females, 50.4%	35–54 years (middle age participants) 271 participants, 45.7%	5–10 years 170, 28.7%	Africa 108, 18.2%
	More than 54 years (old participants) 29 participants, 4.9%	11–15 years 58, 9.8%	Europe 133, 22.4%
		16–20 years 49, 8.3%	North America 33, 5.6%
		More than 21 years 44, 7.4%	South America 8, 1.3%
			Australia 6, 1.0%
Overall 593 participants, 100%	Overall 593 participants, 100%	Overall 593 participants, 100%	Overall 593 participants, 100%

### Attendees' memorising-related factors

The results showed that one of the most important factors that could help memorising the lecture's salient points was the availability of post-VOLSs recordings (62.56%), with a significant difference (*p* = 0.048) depending on the participants residency. The opportunity for having an accessible post-VOLS recording was highly requested by the participants (8.84/10, 95% CI 8.67–9.00) with no significant influence of demographics on this trend (*p* > 0.05). On the other hand, interactive polling throughout VOLSs was the least influential factor (21.08%), with a significant difference (*p* = 0.048) between participants of different age groups. Another factor that was tested in this study as a model for optimising memorising during VOLSs learning, was the taking of digital records (screenshots) by the attendees during the sessions. The average number of screenshots taken was 6.1 per lecture, with a significant impact (*p* = 0.003) of gender on this trend (5.7 by males, 6.5 by females). The continent of attendees also had a significant effect (*p* = 0.016) as those from Australia took an average of 3.7 screenshots per VOLS compared with those from Asia who took 6.4 screenshots per lecture. These screenshots were an additional reading resource for the majority (89.88%).

### Attendees’ motivation-related factors

A deep examination of the data revealed that the learners’ interests in attending VOLSs were mainly for learning new clinical tips (96.8%), getting exposed to evidence-based orthodontics (80.1%), learning teaching style (43.7%), social networking (21.1%), or obtaining a certificate (20.1%). Females were significantly more motivated to learn evidence-based orthodontics compared with males (*p* = 0.031). The continent of residence had a significant effect on the social networking driving factor (*p* = 0.028) with the highest value for Africa (9.8%) and the lowest value for South America (0%) (Appendix 2).

Participants stated that the primary reason for requesting a certificate of attendance was for professional goals (77.23%).The continent of residence have a significant effect on this rationalisation (*p* < 0.05) with a highest value for Europe (64.9%). Likewise, age had a significant effect on these reasonings (*p* < 0.05) with more requests by the younger participants (62.7%) (Appendix 2). When participants were questioned about their favoured method for receiving an attendance certificate, 75.38% preferred to receive it via email, while only a few demanded a copy to be sent by post (2.19%), with no significant influence of demographics on this aspect (*p* > 0.05).

### Attendees’ engagement-related factors

The outcomes revealed that post-lecture feedback played a vital role in reshaping future online activities and increased the rapport between organisers, speakers, and attendees (6.79/10 95% CI 6.58–6.99). Most respondents (7.19/10, 95% CI 6.99–7.39) stated that they responded to the feedback comprehensively and constructively. The results showed that those from Asia (*p* < 0.05), and in particular those younger than 35 years of age (*p* = 0.039), participated more in post-lecture feedback. The majority of participants preferred either very short surveys (46.37%) or short surveys (44.69%), compared with medium (7.42%) or long surveys (1.52%).

## Discussion

Because the live online teaching model has become commonplace in providing educational content during the COVID-19 pandemic [4, 6, 18–21], this study was initiated to best understand the issues concerning conducting of VOLSs. In other parts of this series of articles, we looked at pre-webinar settings during the registration process, and technical settings when attending VOLSs. This part focuses on identifying attendee- and speaker-related issues that could optimise learning and uptake from the VOLSs.

The availability of a recording after a VOLSs was deemed valuable for the reinforcement of the presented material for the participants. This is supported by the findings of previous studies. [22, 23] Though polling questions served in improving participant knowledge and information retention [23–25], our study attested to the fact that interactive polling during the webinars appeared to be of secondary importance, though this trend varied among participants of different ages.

A screenshot is an image of the data displayed on the screen of an electronic device and is a useful resource for providing a reminder of material to research after the online lecture. The taking of screenshots during the webinar enabled documenting important facets of the lectures by many attendees and was advantageous in remembering noteworthy aspects. In the current study, the average number of screenshots taken per lecture by attendees was in the range of 6 screenshots, depending on age, gender, and country of residence. This virtual culture could have a serious impact on copyright and data protection; consequently, speakers should make the audience aware during the e-housekeeping session about the data protection policy of the VOLSs. It might be suggested that speakers opt to flag permissible materials that can be digitally recorded or supply handouts instead. Nevertheless, this aspect is to a large extent beyond control.

The primary purpose of attending VOLSs appeared to be for the learning of clinical orthodontic tips, followed closely by the desire for exposure to orthodontic evidence-based principles. Female participants appeared to be more interested than males in learning orthodontic evidence-based principles. It was suggested that attending a webinar to develop pedagogy skills is crucial [22]. However, our survey showed that learning new teaching styles was of minor significance. Likewise, networking and certificates were of secondary interest. Those who were interested in receiving certificates thought that these were important for their professional portfolios, with the continent seeming to influence this desire. Other reasons given regarding the need for a certificate of attendance included the use of certificates for social media marketing and the claiming of expenses. The favoured way for receiving a certificate of attendance was via email, as it is an easy, secure, and reliable method of communication between attendees and the organisers.

As expected, feedback from VOLSs was an important aspect in improving the learning environment, and this was in agreement with previous studies [23, 24, 26]. It was notable that participants, particularly younger attendees from Asia, stated that they responded comprehensively to requests for short post-VOLSs feedback; this is an indispensable aspect in producing attendee-centered sessions.

## Conclusion

This global cross-sectional study showed that the short feedback survey and post-webinar recording are helpful for revision and recapping. Moreover, speakers should implement appropriate policies to protect their intellectual property and patient data. At the time of conducting this study, the attendees' major interests were learning clinical orthodontic tips and evidence-based orthodontics. This was complemented by the advantage of obtaining emailed certificates for professional portfolio purposes. Although the response rate was low and the sample unevenly distributed, the cohort was global and representative.

## Data Availability

Data are available on request from corresponding author.

## Appendix 1

**Table:** Questionaries of the survey

Survey question	Condition	Options
What is your gender?	Choose one option only	Male Female
What is your age group?	(Choose one option only)	Under 18 18–24 25–34 35–44 45–54 55–64 65+
In what country do you work?	(Choose one option only)	Collected based on the continent
How long have you been practicing orthodontics?	Free text	Number in years
how important is the feedback survey after an online lecture?	On a scale of 1–10	10 = extremely important, 1 = not important
How likely is it that you comprehensively respond to the feedback survey?	On a scale of 1–10	1 unlikely, 10 highly likely
For the feedback survey after an online lecture, what do you prefer?	You can choose one option only	Very short survey (less than 5 questions) Short survey (5–10 questions) Medium length survey (10–20 questions) Long length survey (more than 20 questions) Can you explain why you have chosen the above option?
Which option can help you in memorising the salient points of the lecture?	You can choose more than one option	5-min summary video of the key information introduced by the speaker Post-webinar podcast of the lecture Post-webinar handout and references provided by the speaker Post-webinar recording Interactive polling during the webinar Can you explain why you have chosen the above option
How important is it to have a post-webinar recording?	On a scale of 1–10	10 = extremely important, 1 = not important
What is the number of screen shots you usually take during an online lecture?	On a scale of 1–10	Number of screenshots
What is your aim in taking screenshots?	You can choose more than one option	To share it with a colleague for further discussion To make a note of references for additional reading To make a note about presented graphs and pictures For social media advertisement Can you explain why you have chosen the above option
When you attend an online lecture, what is your major interest(s)?	You can choose more than one option	Learning new clinical tips Learning new evidence Learning new teaching style Social networking Gaining a certificate Can you explain why you have chosen the above option
Why do you need the certificate of attendance?	You can choose more than one option	For professional portfolio purposes To fulfil my CPD/ CME requirements For social media advertisement To claim expenses and tax return Can you explain why you have chosen the above option
How you do you prefer to receive your certificate	You can choose more than one option	By email By post By a downloadable link I do not mind

## Appendix 2

**Table:** Note: numbers that are Bold, italic, and underlined refer to a significant difference

Dimension		Interests				
		Learning new clinical tips (%)	Learning new evidence (%)	Learning new teaching style (%)	Social networking (%)	Gaining a certificate (%)
General		37.0	30.6	16.7	8.1	7.7
Gender	Male	37.5	29.7	16.0	8.8	8.0
	Female	36.5	31.5	17.4	7.3	7.3
Age	Less than 35	37.3	***31******.******8***	15.2	7.8	8.0
	35–54	36.6	***29******.******1***	18.2	8.6	7.5
	More than 54	38.2	***33******.******8***	17.6	4.4	5.9
Practicing	Less than 5 years	38.1	31.4	15.9	7.9	6.7
	5–10 years	37.0	29.9	17.5	7.7	7.9
	11–15 years	33.3	29.6	17.3	11.1	8.6
	16–20 years	36.3	30.4	15.6	6.7	11.1
	More than 21 years	36.2	30.2	19.0	7.8	6.9
Continent	Asia	35.9	30.3	16.5	***8******.******7***	8.6
	Africa	34.5	28.7	18.6	***9******.******8***	8.5
	Europe	41.2	31.8	16.7	***5******.******0***	5.3
	North America	39.0	34.1	12.2	***8******.******5***	6.1
	South America	44.4	33.3	16.7	***0******.******0***	5.6
	Australia	38.5	38.5	15.4	***7******.******7***	0.0

Dimension		Certificate				
		For professional portfolio purposes (%)	To fulfil my CPD/ CME requirements (%)	For social media advertisement (%)	To claim expenses and tax return (%)	
General		57.9	34.1	5.4	2.5	
Gender	Male	54.9	34.1	***7******.******3***	***3******.******7***	
	Female	61.2	34.1	***3******.******4***	***1******.******3***	
Age	Less than 35	***62******.******7***	***29******.******4***	6.6	1.3	
	35–54	***53******.******7***	***38******.******6***	4.1	3.6	
	More than 54	***48******.******6***	***40******.******5***	5.4	5.4	
Practicing	Less than 5 years	58.3	35.4	4.6	1.7	
	5–10 years	59.6	31.6	6.6	2.2	
	11–15 years	50.0	37.8	8.5	3.7	
	16–20 years	59.7	31.9	2.8	5.6	
	More than 21 years	57.6	33.9	5.1	3.4	
Continent	Asia	***55******.******5***	***37******.******8***	4.9	1.9	
	Africa	***64******.******0***	***24******.******3***	8.1	3.7	
	Europe	***64******.******9***	***26******.******8***	5.4	3.0	
	North America	***39******.******0***	***51******.******2***	4.9	4.9	
	South America	***44******.******4***	***55******.******6***	0.0	0.0	
	Australia	***50******.******0***	***50******.******0***	0.0	0.0	

## Abbreviations

VOLSs: Virtual orthodontic learning sessions
CPD: Continuous professional development
IT: Information technology
